# COVID-19 epidemic lockdown-induced changes of cereals and animal protein foods consumption of Iran population: the first nationwide survey

**DOI:** 10.1186/s41043-022-00310-0

**Published:** 2022-07-19

**Authors:** Bahareh Nikooyeh, Samira Rabiei, Maryam Amini, Delaram Ghodsi, Hamid Rasekhi, Azam Doustmohammadian, Zahra Abdollahi, Mina Minaie, Farzaneh Sadeghi, Tirang R. Neyestani

**Affiliations:** 1 Department of Nutrition Research, National Nutrition and Food Technology Research Institute and Faculty of Nutrition Sciences and Food Technology, Shahid Beheshti University of Medical Sciences, Tehran, Iran; 2 Gastrointestinal and Liver Diseases Research Center (GILDRC), Iran University of Medical Sciences, Tehran, Iran; 3 Community Nutrition Office, Deputy of Health, Iran Ministry of Health and Medical Education, Tehran, Iran; 4 Laboratory of Nutrition Research, National Nutrition and Food Technology Research Institute and Faculty of Nutrition Sciences and Food Technology, Shahid Beheshti University of Medical Sciences, Tehran, Iran

**Keywords:** COVID-19, Lockdown, Animal proteins, Cereals, Households

## Abstract

**Background:**

The COVID-19 epidemic has affected diverse issues of life including economy, health and nutrition. This nationwide study was conducted to evaluate the effects of the epidemic lockdown-induced socio-economic changes on animal source proteins and cereals intakes of Iran population for the first time.

**Methods:**

This was a cross-sectional descriptive-analytical study using a web-based electronic self-administered questionnaire. A questionnaire was designed with the aim of detecting any changes in the dietary pattern of the Iranian household following coronavirus epidemic.

**Results:**

A total of 21,290 households participated in the study. Approximately 33%, 24%, 14.2% and 7% of the households had decreased the weekly consumption of red meat, white meat, eggs and rice/bread, respectively, following COVID-19 epidemic. The results of ordered logistic regression showed that the female-headed households, as compared with male-headed ones, were 30% more likely to decrease their weekly consumption of white meat (OR = 1.3, 95% CI: 1.1–1.5). About 8.6% of the households had reduced all three major dietary sources of animal protein. Close to half of the households (46.9%) who decreased their intake of the animal protein sources had increased their intake of rice or bread, too. The major reasons for these changes were decrease of income and job loss.

**Conclusion:**

In conclusion, animal protein food consumption decreased during epidemic lockdown but the amounts of rice and bread, as the major sources of dietary energy, have increased. These changes, if persist long enough, can seriously affect micronutrient status of the whole population. Early nutritional interventions for needy families are warranted.

## Introduction

The new coronavirus infection and related disease, severe acute respiratory syndrome (SARS) or COVID-19, has now become a global health problem with million cases of morbidity and hundreds of thousands deaths [1, 2]. COVID-19 challenges food security through interfering with food systems and impairing food access, as well [3, 4]. Thus, this newly emerged viral disease has many socio-economic implications [5]. Social distancing and lockdown, though necessary for the protection of the population, have seriously damaged world economy, with minor retails as the main casualties. As a result, incomes of many households reduced dramatically due to massive job loss [5, 6].

The relationship between socioeconomic status (SES) and dietary pattern of household is self-evident. With reducing income the items of food basket would be inevitably adjusted with the available and more affordable resources, with increasing the amount of main energy sources usually at the expense of reduction or sometimes omission of micronutrient and protein rich sources [7]. Some studies have demonstrated that fruits and vegetables are the first line of this sacrificing followed by dairy products (notably milk) and meats which may be accompanied by a concomitant increase in other dietary energy sources like refined sugar, white bread and cereals [8–11]. As dietary sources of animal protein are rich in micronutrients that may not be provided by other food groups in sufficient amounts especially in low income families, these changes may have several health consequences. On the other hand, cereals, notably bread and rice, are the far less expensive staple foods providing the major part of dietary energy in Iran [12, 13]. This nationwide study was, therefore, conducted to evaluate the possible effects of these COVID-19 epidemic lockdown-induced socio-economic changes on animal source proteins and cereals intakes of Iran population using a rapid nationwide IT-based household survey for the first time.

## Methods

### Study design

This was a cross-sectional descriptive-analytical study using a web-based electronic self-administered questionnaire. Several virtual sessions were held to design a questionnaire with the aim of detecting any changes in the dietary pattern of the Iranian household following coronavirus epidemic. The content validity was assured by a panel of seven internal (involved in compilation of the questionnaire) and three external (not involved in the compilation of the questionnaire) nutrition experts. A web link was created (https://panel.rabit.ir/s/c1NEPPXL483.html) and the questionnaire was uploaded. Then, an official letter from the Community Nutrition Office, Deputy of Health, Ir. Ministry of Health (MOH), was submitted to the vice-chancellors in health affairs and the Community Nutrition Offices of the medical universities of all provinces. In this letter, the objectives of the project and the related link were explained and it was requested that the provincial health and nutrition workers to notice the community under their service coverage. In addition, the link was distributed massively to all popular social media networks such as Telegram, WhatsApp and also through the MOH website (corona.research.ac.ir). This phase of survey was conducted from 4 to 25 April 2020, during which Iran was in the coronavirus epidemic lockdown. The protocol of this study has already been fully described elsewhere [14]. To compare variables among provinces with different food security situations, we used the latest national report in which provinces are categorized to food insecure (deprived), semi-secure (semi-deprived) and secure (non-deprived) [15].

### Development of the questionnaire

The comprehensive process of development of the questionnaire can be found elsewhere [14]. Each respondent had to complete the questionnaire on behalf of his/her household. The questionnaires were anonymous to ensure the privacy and independence of participants for giving their responses. Questions were asked about socio-economic (SES) and nutritional status of the household before and during corona epidemic. SES section included data regarding gender, education, and occupation of head of the household, household size, province and region of the residency (urban/rural), presence of high-risk person in the household (under-5 children, pregnant or lactating women, elder), and any changes in household income during the coronavirus epidemic. The presence of a person with the history of COVID-19 within the household was also asked. In the nutrition section, the questions were asked regarding the change of frequency in consumption of the selected food items and the reasons for changing consumption frequency during the epidemic.

### Ethical issues

Completion of the questionnaire was voluntary and anonymous. Furthermore, the respondents were assured about confidentiality of information and privacy. Therefore, the completion of the questionnaire was considered as the consent of the respondent to participate in the study. This study was approved by the Ethics Committee of the National Nutrition and Food Technology Research Institute (IR.SBMU.NNFTRI.REC.1399.066).

### Statistical analysis

The descriptive analysis was conducted to assess the distribution of socio-demographic status among respondents. Ordinal logistic regressions were fitted to examine which factors contributed to changes in frequency of consumption of selected food items. Two outcomes were considered as dependent variables in regression models: 1. Changes of frequency of red and white meats, eggs and rice/bread consumption on a weekly basis (increase vs. no changes vs. decrease) 2. Descriptive amount of decrease in consumption of the given foods (slight reduction vs. reduced by half vs. omitted from household food basket). After the test for overall parallel assumption at 0.05 significance, it was indicated that the overall parallel assumption of models has not been violated.

The sex of household head (male, female), living in urban/rural areas (urban, rural), household size (one to two, three to five, six and more), being high risk member(s) in a household (none, under five years old, pregnant/ lactating, elder, more than one member), occupation of head (employee, freelance, retired, health worker, teacher, driver, other), educational status of head (master and higher, bachelor, associate, diploma, high school, theological education (preacher)), changes in income (no changes, small decrease, half, cut), COVID-19 in family (no, yes) and food security status of the province (secure, semi-secure, deprived) were the independent variables assessed. The categorization of the provinces based on food security was according to the latest available national report [15].

In all analyses, sampling weights were used to account for the complex sampling design and to allow inferences valid for the population. Analyses were performed using Stata version 16.0 (StataCorp LLC). A two-tailed *p* < 0.05 was considered significant.

## Results

A total of 21,290 households were included in the analyses. Table 1 shows the socio-demographic characteristics of respondent households. The mean (95% confidence interval [CI]) of age of household head was 44.7 (44.2, 44.9) years, and the data indicated that 26.2% of the households (weighted percentage) were from rural areas.

**Table 1:** Characteristic of the participant households

Variable	*n* (%)^a^
*Urban/rural*	
Urban Rural	14,191 (73.8) 7099 (26.2)
*Household size*	
1–2 3–5 > 6	2883 (15.7) 16,798 (78.2) 1609 (6.1)
*High risk members in household*	
None Under five years old Pregnant/ lactating Elder More than one member	11,511 (52.6) 4881 (21.8) 660 (2.8) 2110 (0.4) 2128 (8.7)
*Sex of the household head*	
Male Female	19,255 (89.8) 2035 (10.2)
*Occupation of the household head*	
Officer Freelance Retired Health workers Teacher Driver Other	3942 (20.5) 7755 (34.3) 1988 (11.7) 572 (2.7) 715 (3.1) 883 (3.9) 5435 (23.8)
*Education of the household head*	
Under diploma Diploma Associate Bachelor Master/higher Theological (preacher)	7981 (32.6) 5277 (24.1) 1540 (7.3) 4022 (21.2) 2338 (14.4) 132 (0.5)

^a^Percentages are weighted

Data showed approximately 33%, 24%, 14.2% and 7% of the households had decreased the weekly consumption of red meat, white meat, eggs and rice/bread, respectively, following COVID-19 epidemic (Table 2).

**Table 2:** Changes in consumption of red and white meat, eggs, rice/bread intake in household per week after COVID-19 epidemic

Variable	Change of weekly consumption	*n* (%)^a^
Red meat	No changes Decrease Increase	13,234 (63.3) 7350 (32.9) 706 (3.8)
White meat	No changes Decrease Increase	14,794 (70.3) 5468 (24.4) 1028 (5.3)
Egg	No changes Decrease Increase	15,343 (70.8) 3342 (14.2) 2605 (15.0)
Bread and rice	No changes Both increase Both decrease Decrease in rice Increase in rice	7776 (66.2) 1263 (7.9) 1612 (7.0) 2807 (13.5) 1075 (5.4)

^a^Percentages are weighted

The results of ordered logistic regression are presented in Table 3. The dependent variables were changes in consumption frequency of red meat, white meat, eggs and rice/bread following COVID-19 epidemic in the models (increased consumption, no changes or decreased consumption). The results showed that the households whose heads were women, as compared with those with men as heads, were 30% more likely to decrease their weekly consumption of white meat after coronavirus epidemic (OR = 1.3, 95% CI: 1.1–1.5).

**Table 3:** Ordered logistic regression models of changes in weekly consumption of selected food items in COVID-19 epidemic

Variable	Red meat OR (95%CI)	White meat OR (95%CI)	Egg OR (95%CI)	Bread and rice OR (95%CI)
*Sex of household head*				
Male	–	–	–	–
Female	1.15 (0.9, 1.33)	1.3 (1.1, 1.5)	1.09 (0.9, 1.3)	1.02 (0.8, 1.2)
*Urban/Rural*				
Urban	–	–	–	–
Rural	0.8 (0.7, 0.9)	0.95 (0.9, 1.05)	1.08 (0.9, 1.2)	1.18 (1.08, 1.3)
*Household size*				
1–2	–	–	–	–
3–5	1.1 (0.96, 1.2)	0.9 (0.8, 1.06)	0.8 (0.7, 0.9)	0.96 (0.85, 1.09)
> 6	1.2 (1.0, 1.4)	1.07 (0.9, 1.3)	0.98 (0.8, 1.2)	1.05 (0.88, 1.2)
*High risk members*				
No	–	–	–	–
< 5 years old	1.2 (1.07, 1.3)	1.09 (0.9, 1.2)	1.07 (0.9, 1.2)	1.01 (0.9, 1.12)
Pregnant/lactating mothers	1.2 (0.9, 1.5)	1.3 (1.03, 1.5)	1.2 (0.9, 1.56)	1.12 (0.89, 1.4)
Elder	1.1 (0.9, 1.3)	1.1 (0.9, 1.2)	0.97 (0.84, 1.1)	1.15 (1.0, 1.32)
More than one	1.4 (1.2, 1.6)	1.5 (1.3, 1.7)	1.3 (1.14, 1.5)	1.07 (0.94, 1.23)
*Occupation*				
Employee	–	–	–	–
Freelance	0.6 (0.5, 0.7)	0.6 (0.5, 0.7)	0.8 (0.7, 0.93)	0.88 (0.75, 1.01)
Retired	1.1 (0.9, 1.3)	1.14 (0.9, 1.3)	1.15 (0.97, 1.4)	1.13 (0.9, 1.35)
Health workers	0.8 (0.6, 1.1)	0.9 (0.7, 1.2)	1.16 (0.9, 1.5)	1.03 (0.8, 1.35)
Teacher	1.4 (1.1, 1.8)	1.4 (1.08, 1.8)	1.13 (0.8, 1.46)	1.43 (1.16, 1.7)
Driver	0.8 (0.7, 1.1)	0.9 (0.7, 1.23)	0.75 (0.58, 0.9)	1.27 (1.01, 1.6)
Other	0.9 (0.7, 1.06)	0.9 (0.8, 1.1)	1.05 (0.9, 1.23)	1.28 (1.1, 1.49)
*Change in income*				
No changes	–	–	–	–
Low decrease	1.5 (1.3, 1.6)	1.5 (1.3, 1.6)	1.19 (1.07, 1.3)	1.16 (1.04, 1.3)
Half	3.7 (3.3, 4.1)	3.5 (3.07, 3.9)	1.6 (1.42, 1.8)	2.09 (1.85, 2.3)
Cut	6.3 (5.6, 7.2)	7.3 (6.4, 8.3)	2.76 (2.39, 3.2)	3.86 (3.4, 4.41)
*COVID-19 in family*				
No	–	–	–	–
Yes	0.9 (0.7, 1.1)	0.8 (0.7, 1.12)	0.84 (0.7, 1.08)	0.8 (0.63, 1.02)
*Education*				
Master./ higher	–	–	–	–
Bachelor	1.4 (1.2, 1.6)	1.5 (1.2, 1.7)	1.24 (1.06, 1.5)	1.14 (0.9, 1.34)
Associate	1.8 (1.5, 2.3)	1.7 (1.4, 2.1)	1.5 (1.2, 1.82)	1.2 (1.01, 1.5)
Diploma	1.8 (1.5, 2.2)	1.7 (1.4, 2.0)	1.9 (1.6, 2.26)	1.3 (1.13, 1.6)
High school	2.2 (1.8, 2.6)	2.1 (1.7, 2.5)	2.48 (2.1, 2.9)	1.6 (1.34, 1.9)
Theological education	2.7 (1.7, 4.4)	3.4 (2.2, 5.6)	7.2 (4.4, 11.6)	3.4 (2.1, 5.4)
*Food security status of province*				
Secure	–	–	–	–
Semi secure	1.3 (1.1, 1.4)	1.1 (0.9, 1.2)	1.37 (1.2, 1.54)	1.2 (1.07, 1.33)
Deprived	1.2 (1.1, 1.4)	1.4 (1.2, 1.6)	1.86 (1.6, 2.1)	1.3 (1.2, 1.47)

Living in rural areas was not a predictor of changing the intake of the selected food items. However, household size was a determinant as the households with more than six members were more likely to decrease their weekly red meat intake compared with households with 1 to 2 members (OR = 1.2, 95% CI: 1.0–1.4).

The households from deprived provinces, compared with those residing in secure provinces, were 20%, 40%, 86% and 30% more likely to decrease their weekly consumption of red meat, white meat, eggs and rice/bread, respectively. There was a significant association between household head’s educational level and changes in weekly consumption pattern of the selected food items during the epidemic.

It is noteworthy that in the subgroup of households that had decreased their weekly consumption of animal protein rich foods and cereals, about 34% (11.2% of total population), 19% (4.7% of total population), 12% (1.7% of total population) and 6% (0.5% of total population) completely omitted red meat, white meat, egg and rice/bread, respectively (Table 4).

**Table 4:** The descriptive amount of decrease in consumption of red and white meats, eggs and rice/bread in the subgroup of households that had decreased their weekly consumption frequency during COVID-19 epidemic lockdown

Food item	Changes status*	*n* (%)	Of total population (%)
Red meat	Little	2296 (32.5)	10.7
	Half	2401 (33.2)	10.9
	Omitted	2653 (34.1)	11.2
White meat	Little	2093 (38.3)	9.3
	Half	2339 (42.2)	10.3
	Omitted	1036 (19.4)	4.7
Egg	Little	1422 (42.5)	6.0
	Half	1521 (45)	6.3
	omitted	399 (12.4)	1.7
Bread and rice	Little	670 (42.8)	2.9
	Half	844 (50.8)	3.5
	Omitted	98 (6.2)	0.5

^*^Little: slight reduction in consumption compared to before COVID-19 epidemic, Half: reduce of consumption by half, Omitted: removed from household food basket

The ordinal regression analysis was performed in the subgroup of the households that decreased their dietary intake to identify factors that were associated with the amount of reduction in red and white meats, eggs and rice/bread consumption. The analysis confirmed that the assumption of parallel odds was not violate (*p* = 0.133). Therefore, results are reported for the ordered logistic models in Table 5. The analysis revealed that living in rural areas was associated with more reduction in weekly consumption of red and white meats, eggs and rice/bread intake. Also, people who were living in semi-secure or deprived provinces were more likely to omit their red meat from their food basket (OR, 1.33, 95%CI: 1.14, 1.54, OR, 1.39, 95%CI: 1.17, 1.65, respectively). Occupation but being teacher was not a significant predictor of more reduction in the food items. Interestingly, households whose income was decreased to half or less were more likely to omit their red and white meats, eggs and rice/bread intake.

**Table 5:** Ordered logistic regression models of factors contributed to the amount of reduction in intake of red meat during COVID-19 epidemic lockdown

Variables	Red meat OR (95% CI)	White meat OR (95% CI)	Egg OR (95% CI)	Bread and rice OR (95% CI)
*Sex of household head*				
Male	–	–	–	–
female	1.01 (0.82, 1.24)	1.07 (0.8, 1.4)	1.02 (0.8, 1.4)	1.09 (0.81, 1.5)
*Urban/Rural*				
Urban	–	–	–	–
Rural	1.38 (1.21, 1.57)	1.23 (1.1, 1.4)	1.3 (1.1, 1.6)	1.7 (1.3, 2.2)
*Household size*				
1–2	–	–	–	–
3–5	0.9 (0.77, 1.17)	0.9 (0.7, 1,1)	0.9 (0.7, 1.2)	0.8 (0.6, 1.03)
> 6	1.07 (0.81, 1.41)	0.8 (0.6, 1.1)	1.04 (0.7, 1.5)	0.6 (0.4, 0.9)
*High risk members*				
No	–	–	–	–
< 5 years old	1.16 (1.0, 1.33)	1.1 (0.9, 1.3)	0.9 (0.7, 1.2)	0.9 (0.7, 1.2)
Pregnant/lactating mothers	1.38 (0.97, 1.96)	1.3 (0.9, 1.9)	0.6 (0.4, 1.01)	0.9 (0.5, 1.4)
Elder	0.84 (0.67, 1.04)	0.9 (0.8, 1.3)	1.1 (0.8, 1.5)	0.8 (0.5, 1.1)
More than one	1.25 (1.04, 1.5)	1.3 (1.0, 1.6)	0.98 (0.8, 1.3)	1.1 (0.8, 1.5)
*Occupation*				
Employee	–	–	–	–
Freelance	0.9 (0.72, 1.12)	0.9 (0.6, 1.2)	0.8 (0.6, 1.2)	0.6 (0.4, 0.9)
Retired	0.97 (0.72, 1.25)	1.2 (0.9,1.7)	0.8 (0.5, 1.3)	1.07 (0.6, 1.8)
Health workers	0.9 (0.6, 1.35)	1.0 (0.6, 1.7)	0.9 (0.5, 1.6)	0.7 (0.3, 1.6)
Teacher	1.41 (1.01, 1.96)	1.5 (0.9, 2.3)	0.6 (0.3, 0.9)	1.3 (0.6, 2.9)
Driver	1.12 (0.82, 1.53)	0.9 (0.6, 1.3)	0.6 (0.4, 0.9)	0.7 (0.4, 1.2)
Other	1.21 (0.97, 1.52)	1.2 (0.9, 1.6)	0.8 (0.6, 1.2)	0.9 (0.6, 1.4)
*Change in income*				
No changes	–	–	–	–
Small decrease	0.92 (0.79, 1.01)	0.9 (0.7, 1.1)	1.2 (0.9, 1.7)	0.7 (0.5, 1.04)
Half	1.29 (1.08, 1.53)	1.7 (1.3, 2.1)	1.7 (1.3, 2.2)	1.7 (1.2, 2.5)
Cut	2.87 (2.36, 3.49)	3.5 (2.7, 4.4)	2.5 (1.9, 3.7)	2.6 (1.8, 3.7)
*COVID-19 in family*				
No	–	–	–	–
Yes	1.54 (1.09, 2.17)	1.3 (0.8, 1.9)	0.9 (0.6, 1.5)	1.2 (0.7, 2.2)
*Education*				
Master./ higher	–	–	–	–
Bachelor	1.21 (0.94, 1.56)	1.6 (1.1, 2.2)	1.3 (0.9, 2.0)	1.5 (0.9, 2.5)
Associate	1.3 (0.97, 1.74)	1.7 (1.2, 2.6)	1.4 (0.9, 2.4)	1.7 (0.9, 3.0)
Diploma	1.33 (1.03, 1.73)	1.6 (1.1, 2.2)	1.4 (0.9, 2.1)	1.6 (1.01, 2.6)
High school	1.85 (1.41, 2.41)	2.0 (1.4, 2.8)	1.3 (0.9, 2.0)	2.1 (1.3, 3.3)
Theological education	2.22 (1.3, 3.8)	1.7 (0.8,3.3)	1.02 (0.5, 2.1)	2.9 (1.3, 6.4)
*Food security status of province*				
Secure	–	–	–	–
Semi secure	1.33 (1.14, 1.54)	1.0 (0.8, 1.2)	0.8 (0.6, 1.1)	0.7 (0.5, 0.9)
Deprived	1.39 (1.17, 1.65)	1.1 (0.9, 1.3)	1.04 (0.8, 1.4)	1.08 (0.8, 1.5)

**Fig. 1:**
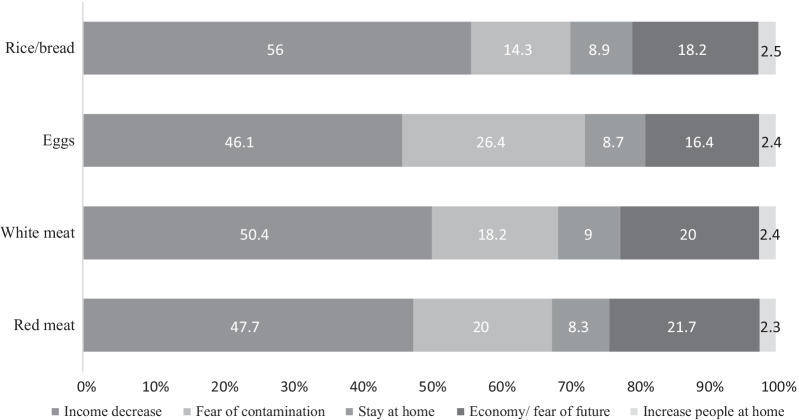
Causes of decrease in weekly consumption frequency of some selected food items during COVID-19 epidemic lockdown in the households that had decreased their intake

**Fig. 2:**
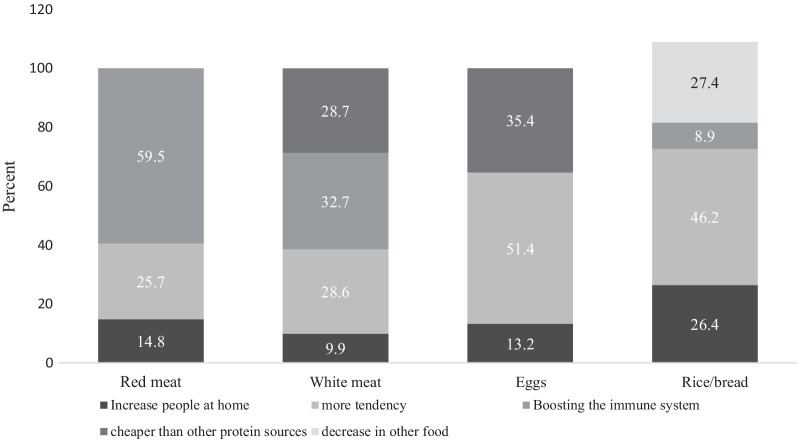
Causes of increased weekly consumption frequency of meats, eggs and rice/bread during COVID-19 epidemic lockdown in the households that had increased their intake

Figures 1 and 2 display the stacked par charts for the reasons of decrease or increase of weekly consumption of the selected food items during the epidemic. The main reason for reduced consumption was income decrement and job loss.

A total of 20.1% of households had decreased weekly consumption of both red and white meats and 8.6% had reduced all three major dietary sources of animal protein, i.e., red and white meats and eggs. Close to half of the households (46.9%) who decreased their intake of the three protein sources had increased their intake of rice or bread.

## Discussion

This is the first nationwide report of the coronavirus epidemic-induced dietary changes of the Iranian households during lockdown period. The mean household size and the ratio of urban to rural households well correspond the report from latest population census in Iran [16] indicating that the study population was representative (~ 1%) of the whole country households.

We found a considerable decrease in consumption frequency of animal protein rich foods notably red meat followed by white meat and eggs during coronavirus epidemic lockdown. The main reason for this change in dietary pattern in our study was reduced income which inevitably affects purchasing power and hence food access. Fear of exposure to asymptomatic carriers of the coronavirus may also adversely affect food choices of the households [17]. On the other hand, reduced demand for certain food items, including animal source proteins, may lead to a substantial decrement in vendors' and retailers' income [17]. Consequently, COVID-19-induced changes of dietary pattern of Iranian households have been, so far, due to decreased economic and physical access. Inaccessibility of food due to costs may result in socioeconomic disparities in healthy diet [7]. Obviously, poor people and those households with bigger size are more vulnerable. Decreased food access, one of the impacts of the epidemic on food security of the households [18], may be accompanied by less healthy eating pattern and poor diet quality [19].

Our findings demonstrated that female headed-households were more vulnerable and more likely to decrease their animal source proteins in their food baskets. Recent studies have addressed this issue that female-headed households confronting several challenges might be more vulnerable than male-headed [20] and strategies imposed by governments to combat coronavirus epidemic may adversely affect food security of the vulnerable subgroups including female-headed households [21].

We found that the likelihood of decrement of animal source proteins from food basket in deprived (food insecure), as compared with secure, provinces is higher. Furthermore, the quality of foods purchased could be very different, usually worse, when the household income decreases [10, 22]. Though we did not really evaluate food security status of the households, decreased animal protein foods in the food basket usually accompanies food insecurity [23].

A very noticeable finding is increment of bread and rice consumption in almost half of the study population. Generally socio-economic status has a direct correlation with diet quality in terms of micronutrient content [24]. It should be noted that high animal protein intake is not generally recommended from both nutrition and environmental points of view [25, 26] and there is evidence for association between animal protein intake and all-cause as well as specific (notably general adiposity and cardiovascular disease) mortality [26–29]. Nevertheless, decreased or deletion of animal source proteins due to reduced access of the households together with consequent increased consumption of cereals, notably rice and bread, may bring about adverse health effects including obesity with its comorbidities such as diabetes, hypertension and cardiovascular disease, with concomitant micronutrient deficiencies, the so-called double burden of malnutrition [30, 31]. High occurrence of micronutrient deficiencies and related disorders including stunting in under 5 children has been partly attributed to insufficient or lack of animal source proteins in diet [32]. Findings from two recent studies confirmed the effect of animal source proteins intake by pregnant mother and growing child in prevention and treatment of different forms of child malnutrition notably underweight, wasting and stunting [33, 34].

Some limitations of this study must be acknowledged. The survey employed self-administered online questionnaire with the consequent exclusion of those people who had less or no access to the internet facilities for any reason. Nevertheless, this is a common limitation in online surveys [35–37]. Furthermore, we did not ask about the household fish consumption. Considering the high price of fish in Iran is one of the biggest obstacles to fish consumption [38], noticeable decrement and even omission of this nutritious food item from the food basket of many Iranian households during the epidemic is highly expectable. Notwithstanding, we do not think that this issue has affected seriously our findings as some studies showed that the contribution of fish to total protein intake of the Iranian households is minimal [39, 40].

## Conclusions

The COVID-19 epidemic has affected different aspects of life including economy, food choices and also food access. Our findings revealed that the contribution of animal protein foods in a considerable proportion of Iranian household food baskets have decreased during epidemic lockdown mostly due to income and job loss but the amounts of rice and bread, as the major sources of dietary energy, have increased. These changes, if last long enough, can seriously affect micronutrient status of the whole population, especially children, and accelerate the occurrence rates of obesity and several chronic diseases in the future. Further research is thus warranted to determine if this trend is secular. Meanwhile, early nutritional interventions including subsidies, food basket aids, home gardening, home fortification and supplementation for needy families should be implemented.

## Data Availability

Please contact author for data requests.

## References

[1] Sohrabi C, Alsafi Z, O’Neill N, Khan M, Kerwan A, Al-Jabir A, Iosifidis C, Agha R (2020). World Health Organization declares global emergency: a review of the 2019 novel coronavirus (COVID-19). Int J Surg.

[2] Lewnard JA, Lo NC (2020). Scientific and ethical basis for social-distancing interventions against COVID-19. Lancet Infect Dis.

[3] Devereux S, Béné C, Hoddinott J (2020). Conceptualising COVID-19’s impacts on household food security. Food Secur.

[4] Deaton BJ, Deaton BJ (2020). Food security and Canada's agricultural system challenged by COVID-19. Can J Agric Econ.

[5] Nicola M, Alsafi Z, Sohrabi C, Kerwan A, Al-Jabir A, Iosifidis C, Agha M, Agha R (2020). The socio-economic implications of the coronavirus pandemic (COVID-19): a review. Int J Surg.

[6] Coibion O, Gorodnichenko Y, Weber M. The cost of the covid-19 crisis: Lockdowns, macroeconomic expectations, and consumer spending. In: National Bureau of Economic Research; 2020.

[7] Darmon N, Drewnowski A (2015). Contribution of food prices and diet cost to socioeconomic disparities in diet quality and health: a systematic review and analysis. Nutr Rev.

[8] Muhammad A, D’Souza A, Meade B, Micha R, Mozaffarian D. The influence of income and prices on global dietary patterns by country, age, and gender. 2017. 10.1136/bmjgh-2016-000184 PMC5717967 29225943

[9] Bonaccio M, Bonanni AE, Di Castelnuovo A, De Lucia F, Donati MB, De Gaetano G, Iacoviello L (2012). Investigators M-sP: Low income is associated with poor adherence to a Mediterranean diet and a higher prevalence of obesity: cross-sectional results from the Moli-sani study. BMJ Open.

[10] French SA, Tangney CC, Crane MM, Wang Y, Appelhans BM (2019). Nutrition quality of food purchases varies by household income: the SHoPPER study. BMC Public Health.

[11] Engler-Stringer R (2011). Food selection and preparation practices in a group of young low-income women in Montreal. Appetite.

[12] Kalantari N, Ghafarpour M. National Comprehensive study on household food consumption pattern and nutritional status, IR Iran, 2001-2003 (National Report). In: Tehran Shahid Beheshti Medical University, National Nutrition and Food Technology Research Institute; 2005.

[13] Rahmani O, Rezania S, Beiranvand Pour A, Aminpour SM, Soltani M, Ghaderpour Y, Oryani B (2020). An Overview of household energy consumption and carbon dioxide emissions in Iran. Processes.

[14] Rasekhi H, Rabiei S, Amini M, Ghodsi D, Doustmohammadian A, Nikooyeh B, Abdollahi Z, Minaie M, Sadeghi F, Neyestani TR (2021). COVID-19 epidemic-induced changes of dietary intake of Iran population during lockdown period: the study protocol national food and nutrition surveillance. Nutr Food Sci Res.

[15] Kolahdooz F, Najafi F. Report of a National Survey. Food security information and mapping system in Iran [Persian]. In: Tehran Iran Ministry of Health, Treatment and Medical Education; 2012.

[16] Iran Statistics Center. The concise report of the nationwide income-espense census of Iranian urban and rural households, 2018. Population, work force and census office (https://www.amar.org.ir/Portals/0/News/1398/1_ch-hvd97.pdf) [In Persian]. In: 2019.

[17] Béné C (2020). Resilience of local food systems and links to food security–a review of some important concepts in the context of COVID-19 and other shocks. Food Secur.

[18] Niles MT, Bertmann F, Belarmino EH, Wentworth T, Biehl E, Neff R (2020). The early food insecurity impacts of COVID-19. Nutrients.

[19] Leung CW, Epel ES, Ritchie LD, Crawford PB, Laraia BA (2014). Food insecurity is inversely associated with diet quality of lower-income adults. J Acad Nutr Diet.

[20] Yoosefi Lebni J, Mohammadi Gharehghani MA, Soofizad G, Khosravi B, Ziapour A, Irandoost SF (2020). Challenges and opportunities confronting female-headed households in Iran: a qualitative study. BMC Womens Health.

[21] Crush J, Si Z (2020). COVID-19 containment and food security in the Global South. J Agric Food Syst Community Dev.

[22] Imamura F, Micha R, Khatibzadeh S, Fahimi S, Shi P, Powles J, Mozaffarian D, Nutrition GBoD, Group CDE (2015). Dietary quality among men and women in 187 countries in 1990 and 2010: a systematic assessment. Lancet Glob Health.

[23] Rodríguez-Pérez C, Molina-Montes E, Verardo V, Artacho R, García-Villanova B, Guerra-Hernández EJ, Ruíz-López MD (2020). Changes in dietary behaviours during the COVID-19 outbreak confinement in the Spanish COVIDiet study. Nutrients.

[24] Novaković R, Cavelaars A, Geelen A, Nikolić M, Altaba II, Vinas BR, Ngo J, Golsorkhi M, Medina MW, Brzozowska A (2014). Review article socio-economic determinants of micronutrient intake and status in Europe: a systematic review. Public Health Nutr.

[25] Tilman D, Clark M (2014). Global diets link environmental sustainability and human health. Nature.

[26] Song M, Fung TT, Hu FB, Willett WC, Longo VD, Chan AT, Giovannucci EL (2016). Association of animal and plant protein intake with all-cause and cause-specific mortality. JAMA Intern Med.

[27] Fung TT, van Dam RM, Hankinson SE, Stampfer M, Willett WC, Hu FB (2010). Low-carbohydrate diets and all-cause and cause-specific mortality: two cohort studies. Ann Intern Med.

[28] Chen Z, Glisic M, Song M, Aliahmad HA, Zhang X, Moumdjian AC, Gonzalez-Jaramillo V, van der Schaft N, Bramer WM, Ikram M (2020). Dietary protein intake and all-cause and cause-specific mortality: results from the Rotterdam study and a meta-analysis of prospective cohort studies. Eur J Epidemiol.

[29] Segovia-Siapco G, Khayef G, Pribis P, Oda K, Haddad E, Sabaté J (2020). Animal protein intake is associated with general adiposity in adolescents: the teen food and development study. Nutrients.

[30] Dasi T, Selvaraj K, Pullakhandam R, Kulkarni B (2019). Animal source foods for the alleviation of double burden of malnutrition in countries undergoing nutrition transition. Anim Front.

[31] Seligman HK, Laraia BA, Kushel MB (2010). Food insecurity is associated with chronic disease among low-income NHANES participants. J Nutr.

[32] Adesogan AT, Havelaar AH, McKune SL, Eilittä M, Dahl GE (2020). Animal source foods: sustainability problem or malnutrition and sustainability solution. Perspective matters. Global Food Secur.

[33] Pimpin L, Kranz S, Liu E, Shulkin M, Karageorgou D, Miller V, Fawzi W, Duggan C, Webb P, Mozaffarian D (2019). Effects of animal protein supplementation of mothers, preterm infants, and term infants on growth outcomes in childhood: a systematic review and meta-analysis of randomized trials. Am J Clin Nutr.

[34] Kaimila Y, Divala O, Agapova SE, Stephenson KB, Thakwalakwa C, Trehan I, Manary MJ, Maleta KM (2019). Consumption of animal-source protein is associated with improved height-for-age z scores in rural Malawian children aged 12–36 months. Nutrients.

[35] de Melo AF, Homem-de-Mello M (2020). High-dose intravenous vitamin C may help in cytokine storm in severe SARS-CoV-2 infection. Crit Care.

[36] Litton MM, Beavers AW (2021). The relationship between food security status and fruit and vegetable intake during the COVID-19 pandemic. Nutrients.

[37] Malta DC, Szwarcwald CL (2020). Barros MBdA, Gomes CS, Machado ÍE, Souza Júnior PRBd, Romero DE, Lima MG, Damacena GN, Pina MdF: The COVID-19 Pandemic and changes in adult Brazilian lifestyles: a cross-sectional study, 2020. Epidemiol Serv Saude.

[38] Rezaeipandari H (2014). Barriers to fish consumption and it’s influencing factors: a comprehensive overview of the relevant evidence in Iran and in the world. J Health Field.

[39] Adeli A (2013). Fish consumption in Iran and the world. J Mar Sci Technol Res.

[40] Dadgar S, Haji Mirmohammadi SD, Hafezieh M, Teimouri M, Nekouifard A, Seidgar M, Sharifian M (2021). Per capita fish consumption and the factors affecting It in West Azerbaijan province Iran. Util Cultiv Aquat.

